# Crystal structure and Hirshfeld surface analysis of 6,6′-((1*E*,1′*E*)-{[1,4-phenyl­enebis(methyl­ene)]bis(aza­nylyl­idene)}bis­(methane­ylyl­idene))bis­(2-meth­oxy­phenol)

**DOI:** 10.1107/S2056989021013347

**Published:** 2022-01-01

**Authors:** Sevgi Kansiz, Semanur Yesilbag, Necmi Dege, Eiad Saif, Erbil Agar

**Affiliations:** a Samsun University, Faculty of Engineering, Department of Fundamental Sciences, 55420, Samsun, Turkey; b Department of Chemistry, Faculty of Arts and Sciences, Ondokuz Mayıs University, 55139, Samsun, Turkey; c Ondokuz Mayıs University, Faculty of Arts and Sciences, Department of Physics, 55139, Samsun, Turkey; d Department of Computer and Electronic Engineering Technology, Sanaa Community, College, Sanaa, Yemen; e Ondokuz Mayıs University, Faculty of Engineering, Department of Electrical and Electronic Engineering, 55139, Samsun, Turkey; f Ondokuz Mayıs University, Faculty of Arts and Sciences, Department of Chemistry, 55139, Samsun, Turkey

**Keywords:** crystal structure, tetra­dentate salen-type, Schiff base, phenol–imine tautomer, Hirshfeld surface analysis

## Abstract

The Schiff base compound, C_24_H_24_N_2_O_4_, was synthesized by the inter­action of 2-hy­droxy-3-meth­oxy benzaldehyde and 1,4-benzene dimethanamine in ethanol, and crystallizes in the monoclinic space group *P*2_1_/*n* with *Z*′ = 0.5. The mol­ecule is not planar, the 1,4-di­ethyl­benzene and the phenol rings are twisted with respect to each other, making a dihedral angle of 74.27 (5)°. The mol­ecular structure is stabilized by an O—H⋯N hydrogen bond, forming an *S*(6) ring motif. In the crystal, mol­ecules are linked by C—H⋯O hydrogen bonds, resulting in the formation of sheets parallel to the *bc* plane. A Hirshfeld surface analysis was undertaken to investigate the various inter­molecular contacts controlling the supra­molecular topology, suggesting the H⋯O (18%) contacts to be the most significant inter­actions, whereas the H⋯H (50.5%) and C⋯H (24.3%) inter­actions are less significant.

## Abstract 2

The title compound is a Schiff base that exists in the phenol–imine tautomeric form. The mol­ecular structure is stabilized by an O—H⋯N hydrogen bond, forming an *S*(6) ring motif.

## Chemical context

Schiff bases are used as pigments and dyes, catalysts, inter­mediates in organic synthesis, and as polymer stabilizers (Supuran *et al.*, 1996 ▸). In azomethine derivatives, the C=N linkage is essential for biological activity and several azo­methines have been reported to possess remarkable anti­bacterial, anti­fungal, anti­cancer and diuretic activities (Gaur, 2003 ▸). Schiff bases having an azomethine group of general formula C=N– contain various substituted groups (Schiff, 1864 ▸). Of particular inter­est are the two different tautomeric structures for *o*-hy­droxy Schiff bases, which are expressed as keto-amine and phenol-imine, with intrinsic N—H⋯O or O—H⋯N hydrogen bonds, (Filarowski *et al.*, 2004 ▸). There are many studies in the literature on the synthesis of Schiff bases and investigation of tautomeric structures. Phenol-imine and keto-amine tautomeric structures exhibit features of photochromism and thermochromism (Hadjoudis *et al.*, 2004 ▸). Tetra­dentate salen-type ligands have been used in almost all areas of coordination chemistry to prepare complexes that have catalytic and biological activity or which feature inter­esting structural, electrochemical or magnetic properties (Abd El-Hamid *et al.*, 2019 ▸). In this study, a symmetrical tetra­dentate Schiff base ligand bearing ONNO donor atoms, 6,6′-((1*E*,1′*E*)-{[1,4-phenyl­enebis(methyl­ene)]bis­(aza­nylylidene)}bis­(methane­ylyl­idene))bis­(2-meth­oxy­phenol) was synthesized by the inter­action of 2-hy­droxy-3-meth­oxy benzaldehyde and 1,4-benzene dimethanamine in ethanol and its crystal structure determined by single-crystal X-ray diffraction. 
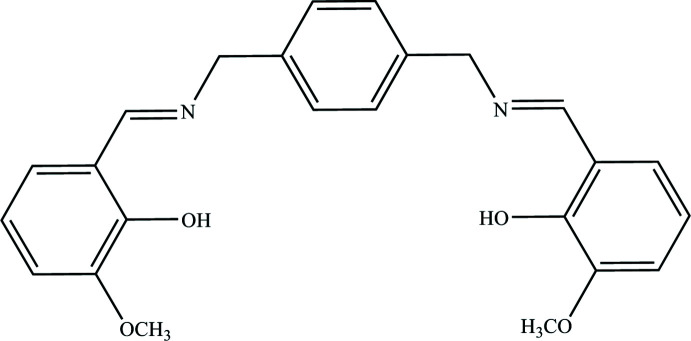


## Structural commentary

**Figure 1:**
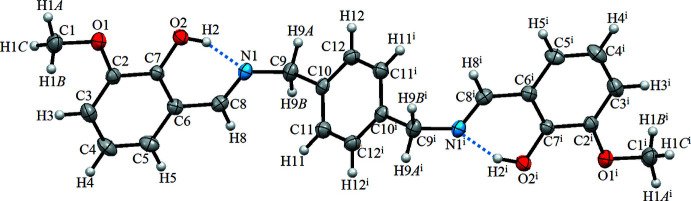
The mol­ecular structure of the title compound with displacement ellipsoids drawn at the 40% probability level. Symmetry code: (i) −*x* + 2, −*y* + 1, −*z* + 1.

The mol­ecular structure of the title Schiff base derivative is illustrated in Fig. 1 ▸. The asymmetric unit of the title compound contains one-half of the centrosymmetric mol­ecule (*Z*′ = 0.5). There is an intra­molecular O2—H2⋯N1 hydrogen bond (Table 1 ▸ and Fig. 1 ▸); this is a common feature also observed in related phenol-imine Schiff bases. It forms an *S*(6) ring motif and also induces the phenol ring and the Schiff base to be nearly coplanar, as indicated by the C6—C8—N1—C9 torsion angle of 178.54 (13)°. The mol­ecule is non-planar, the 1,4-di­ethyl­benzene ring being inclined to the phenol ring by 74.27 (5)°. The C7—C6—C8—N1 torsion angle [3.8 (2)°] further supports the co-planarity of the phenol ring and the Schiff base. The C7—O2 distance is 1.3438 (17) Å, which is close to normal values reported for single C—O bonds in phenols and salicyl­idene­amines (Kaştaş & Albayrak Kaştaş, 2019 ▸). The N1—C8 bond is short at 1.2717 (17) Å, strongly indicating a C=N double bond, while the long C6—C8 bond [1.451 (2) Å] implies a single bond. All of these data support the existence of the phenol–imine tautomer for the title compound in the crystalline state.

## Supra­molecular features

**Figure 2:**
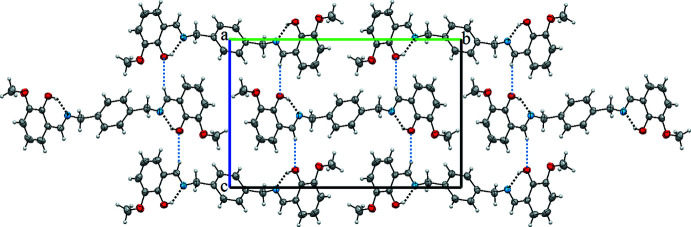
A view of the crystal packing of the title compound in a view parallel to the *bc* plane. C—H⋯O hydrogen bonds are shown as dashed blue lines.

In the crystal, mol­ecules are connected into sheets extending in the *bc* plane by C8—H8⋯O2^i^ hydrogen bonds (Table 1 ▸; Fig. 2 ▸).

## Database survey

A search of the Cambridge Structural Database (CSD Version 5.42, update of May 2021; Groom *et al.*, 2016 ▸) for the (1,4-phenyl­ene)bis­(*N*-ethyl­idenemethanamine) moiety revealed some related structures. The most similar structures are 1,4-bis­(2-pyridyl­methyl­ene­amino­meth­yl)benzene (GOLJUN; Li *et al.*, 2009 ▸), 1,4-bis­(3-pyridyl­methyl­ene­amino­meth­yl)benzene (GOLJOH; He *et al.*, 2009 ▸) and 1,4-bis­(3,5-di-*t*-butyl-2-hy­droxy­benzyl­idene­amino­meth­yl)benzene (OCAPAK; Tooke *et al.*, 2004 ▸). In GOLJUN and GOLJOH, the mol­ecules have similar shapes to the title compound. The C—N bond lengths [1.253 (2) Å in GOLJOH and 1.256 (2) Å in GOLJUN] are typical for an azomethine C=N bond and shorter than in the title compound [1.2717 (19) Å]. The torsion angles involving the C—C=N—C units are −177.26 (11)° and 115.21 (13)° in GOLJUN. These values are similar to those observed in the title compound. In OCAPAK, a *t*-butyl group is present, different from the title compound. In addition, there is an intra­molecular O—H⋯N contact in the title compound. Similarly, in OCAPAK, the hydroxyl H atom is involved in an intra­molecular O—H⋯N hydrogen bond, forming an *S*(6) ring motif as in the title compound. The length of intra­molecular O—H⋯N hydrogen bond in OCAPAK is especially short [1.65 (2) Å] compared to that in the title compound [1.789 (15) Å].

## Hirshfeld surface analysis

**Figure 3:**
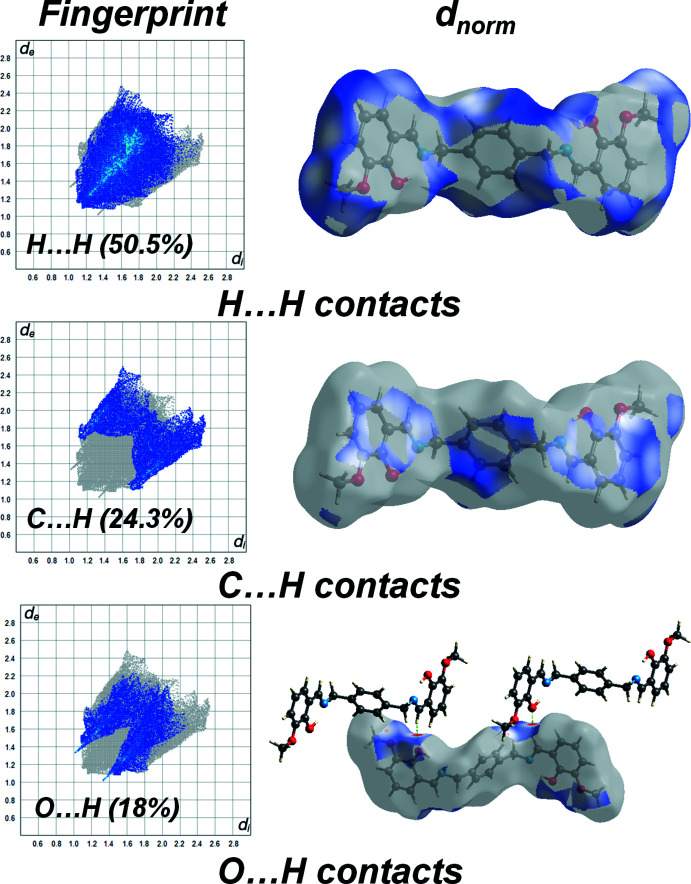
The Hirshfeld surface analysis of the title compound mapped with *d*_norm_ over −0.175 to 1.404 a.u. showing the C—H⋯O hydrogen-bonded contacts.

Hirshfeld surface analysis was used to analyse the various inter­molecular inter­actions in the title compound, through mapping of the normalized contact distance (*d*_norm_) using *CrystalExplorer17* (Turner *et al.*, 2017 ▸; Spackman & Jayatilaka, 2009 ▸). Hirshfeld surface analysis is a valuable tool for assessing the strength of inter­molecular inter­actions, predicting the properties of a crystal and its potential applications (Al-Resayes *et al.*, 2020 ▸). The Hirshfeld surface was generated using a standard (high) surface resolution with the three-dimensional *d*_norm_ surface mapped over a fixed color scale of −0.175 (red) to 1.404 a.u. (blue). The packing of mol­ecules is mainly dependent on H⋯H (50.5%) and C⋯H (24.3%) inter­actions and the significant C—H⋯O inter­actions (18%). Blue regions in the *d*_norm_ map indicate inter­molecular inter­actions with distances longer than van der Waals radius sum of the inter­acting elements (Fig. 3 ▸). The C—H⋯O inter­actions, which appear as red spots in the *d*_norm_ map, have contact distances shorter than the sum of the van der Waals radii of the oxygen and hydrogen atoms

## Synthesis and crystallization

**Figure 4:**
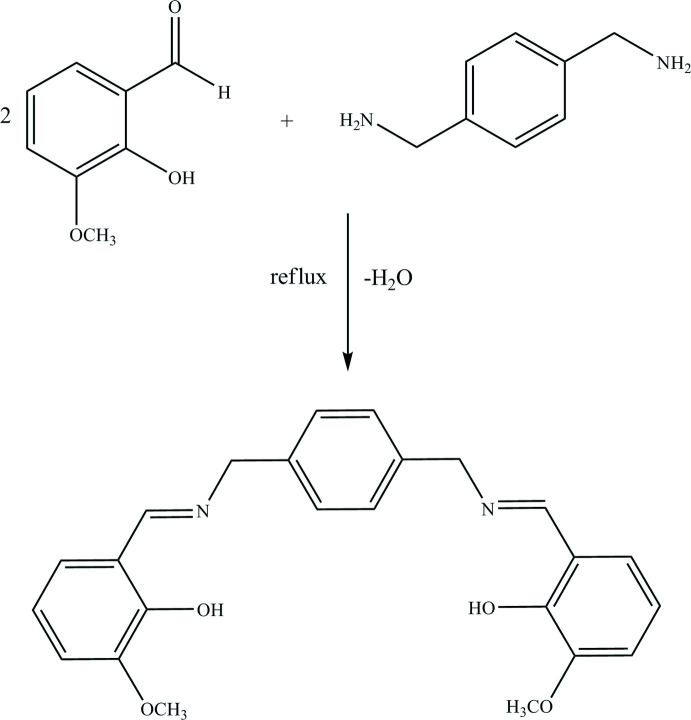
The synthesis of the title compound.

0.0225 g (0.148 mmol) of 2-hy­droxy-3-meth­oxy benzaldehyde was dissolved in 20 mL of ethanol and mixed with 0.0100 g (0.074 mmol) of 1,4-benzene dimethanamine dissolved in 20 mL of ethanol (Fig. 4 ▸). The reaction mixture was refluxed for 6 h and at the end of the reaction, the solution was allowed to cool. The yellow product obtained was washed with ether and crystallized in ethanol at room temperature (m.p. = 431–434 K, yield 85%).

## Refinement

Crystal data, data collection and structure refinement details are summarized in Table 2 ▸. The O–bound H atom was located in a difference-Fourier map and refined with with *U*_iso_(H) = 1.5*U*_eq_(O) and a distance restraint. The C-bound H atoms were positioned geometrically (C—H = 0.93, 0.96 and 0.97 Å) and refined using a riding model, with *U*_iso_(H) = 1.5*U*_eq_(C) for methyl H atoms and 1.2*U*_eq_(C) for other H atoms.

## Supplementary Material

Crystal structure: contains datablock(s) I. DOI: 10.1107/S2056989021013347/jq2011sup1.cif

Structure factors: contains datablock(s) I. DOI: 10.1107/S2056989021013347/jq2011Isup2.hkl

Supporting information file. DOI: 10.1107/S2056989021013347/jq2011Isup3.cml

CCDC reference: 2128953

Additional supporting information:  crystallographic information; 3D view; checkCIF report

## Figures and Tables

**Table 1:** Hydrogen-bond geometry (Å, °)

*D*—H⋯*A*	*D*—H	H⋯*A*	*D*⋯*A*	*D*—H⋯*A*
O2—H2⋯N1	0.86 (1)	1.79 (2)	2.5877 (18)	154 (2)
C8—H8⋯O2^i^	0.93	2.51	3.410 (2)	162

Symmetry code: (i) x+{\script{1\over 2}}, -y+{\script{1\over 2}}, z+{\script{1\over 2}}.

**Table 2:** Experimental details

Crystal data	
Chemical formula	C_24_H_24_N_2_O_4_
*M*_r_	404.45
Crystal system, space group	Monoclinic, *P*2_1_/*n*
Temperature (K)	296
*a*, *b*, *c* (Å)	4.7339 (10), 18.406 (4), 11.880 (2)
β (°)	98.47 (3)
*V* (Å^3^)	1023.8 (4)
*Z*	2
Radiation type	Mo *K*α
μ (mm^−1^)	0.09
Crystal size (mm)	0.61 × 0.45 × 0.23

Data collection	
Diffractometer	Stoe IPDS 2
Absorption correction	Integration (*X-RED32*; Stoe & Cie, 2002 ▸)
*T*_min_, *T*_max_	0.963, 0.992
No. of measured, independent and observed [*I* > 2σ(*I*)] reflections	6766, 1863, 1315
*R*_int_	0.034
(sin θ/λ)_max_ (Å^−1^)	0.600

Refinement	
*R*[*F* ^2^ > 2σ(*F* ^2^)], *wR*(*F* ^2^), *S*	0.037, 0.092, 1.01
No. of reflections	1863
No. of parameters	140
No. of restraints	1
H-atom treatment	H atoms treated by a mixture of independent and constrained refinement
Δρ_max_, Δρ_min_ (e Å^−3^)	0.11, −0.09

Computer programs: *X-AREA* and *X-RED* (Stoe & Cie, 2002 ▸), *SHELXT2014/5* (Sheldrick, 2015*a* ▸), *SHELXL2018/3* (Sheldrick, 2015*b* ▸) and *PLATON* (Spek, 2020 ▸).

## supplementary crystallographic information

### Crystal data

**Table:**

C_24_H_24_N_2_O_4_	*F*(000) = 428
*M**_r_* = 404.45	*D*_x_ = 1.312 Mg m^−^^3^
Monoclinic, *P*2_1_/*n*	Mo *K*α radiation, λ = 0.71073 Å
*a* = 4.7339 (10) Å	Cell parameters from 7423 reflections
*b* = 18.406 (4) Å	θ = 1.7–31.5°
*c* = 11.880 (2) Å	µ = 0.09 mm^−^^1^
β = 98.47 (3)°	*T* = 296 K
*V* = 1023.8 (4) Å^3^	Plate, yellow
*Z* = 2	0.61 × 0.45 × 0.23 mm

### Data collection

**Table:**

Stoe IPDS 2 diffractometer	1863 independent reflections
Radiation source: sealed X-ray tube, 12 x 0.4 mm long-fine focus	1315 reflections with *I* > 2σ(*I*)
Detector resolution: 6.67 pixels mm^-1^	*R*_int_ = 0.034
rotation method scans	θ_max_ = 25.3°, θ_min_ = 2.1°
Absorption correction: integration (X-RED32; Stoe & Cie, 2002)	*h* = −5→5
*T*_min_ = 0.963, *T*_max_ = 0.992	*k* = −22→22
6766 measured reflections	*l* = −13→14

### Refinement

**Table:**

Refinement on *F*^2^	Primary atom site location: structure-invariant direct methods
Least-squares matrix: full	Secondary atom site location: difference Fourier map
*R*[*F*^2^ > 2σ(*F*^2^)] = 0.037	Hydrogen site location: mixed
*wR*(*F*^2^) = 0.092	H atoms treated by a mixture of independent and constrained refinement
*S* = 1.01	*w* = 1/[σ^2^(*F*_o_^2^) + (0.049*P*)^2^] where *P* = (*F*_o_^2^ + 2*F*_c_^2^)/3
1863 reflections	(Δ/σ)_max_ < 0.001
140 parameters	Δρ_max_ = 0.11 e Å^−^^3^
1 restraint	Δρ_min_ = −0.09 e Å^−^^3^

### Special details

**Table:**

Geometry. All esds (except the esd in the dihedral angle between two l.s. planes) are estimated using the full covariance matrix. The cell esds are taken into account individually in the estimation of esds in distances, angles and torsion angles; correlations between esds in cell parameters are only used when they are defined by crystal symmetry. An approximate (isotropic) treatment of cell esds is used for estimating esds involving l.s. planes.

### Fractional atomic coordinates and isotropic or equivalent isotropic displacement parameters (Å^2^)

**Table:**

	*x*	*y*	*z*	*U*_iso_*/*U*_eq_	
O2	0.6966 (3)	0.21666 (6)	0.38262 (9)	0.0633 (3)	
H2	0.817 (4)	0.2501 (10)	0.4060 (16)	0.095*	
O1	0.3093 (3)	0.11352 (6)	0.35977 (10)	0.0722 (4)	
N1	1.0411 (3)	0.30049 (6)	0.51303 (11)	0.0550 (3)	
C7	0.6181 (3)	0.18967 (7)	0.47846 (12)	0.0499 (4)	
C6	0.7362 (3)	0.21470 (8)	0.58611 (12)	0.0528 (4)	
C10	1.1273 (3)	0.43140 (8)	0.51623 (13)	0.0533 (4)	
C8	0.9571 (3)	0.26998 (8)	0.59825 (13)	0.0561 (4)	
H8	1.040345	0.283593	0.671014	0.067*	
C2	0.4103 (3)	0.13480 (8)	0.46848 (13)	0.0560 (4)	
C12	1.1668 (4)	0.47353 (9)	0.42431 (14)	0.0620 (4)	
H12	1.279589	0.456251	0.372334	0.074*	
C9	1.2603 (4)	0.35690 (8)	0.53440 (15)	0.0629 (4)	
H9A	1.400552	0.350226	0.483419	0.075*	
H9B	1.357488	0.352833	0.611939	0.075*	
C11	0.9593 (4)	0.45861 (9)	0.59194 (14)	0.0634 (4)	
H11	0.930241	0.431002	0.654796	0.076*	
C5	0.6416 (4)	0.18568 (10)	0.68255 (14)	0.0697 (5)	
H5	0.719087	0.202247	0.754451	0.084*	
C3	0.3230 (4)	0.10710 (9)	0.56514 (17)	0.0702 (5)	
H3	0.186333	0.070445	0.558980	0.084*	
C4	0.4365 (4)	0.13326 (11)	0.67187 (16)	0.0786 (5)	
H4	0.372061	0.114863	0.736433	0.094*	
C1	0.0961 (4)	0.05885 (10)	0.34579 (18)	0.0816 (6)	
H1A	0.044148	0.048311	0.266297	0.122*	
H1B	−0.068976	0.075560	0.376457	0.122*	
H1C	0.168648	0.015654	0.385164	0.122*	

### Atomic displacement parameters (Å^2^)

**Table:**

	*U*^11^	*U*^22^	*U*^33^	*U*^12^	*U*^13^	*U*^23^
O2	0.0715 (8)	0.0674 (7)	0.0513 (6)	−0.0180 (6)	0.0096 (5)	0.0000 (5)
O1	0.0729 (8)	0.0703 (7)	0.0742 (8)	−0.0230 (6)	0.0139 (6)	−0.0124 (6)
N1	0.0550 (8)	0.0453 (7)	0.0626 (8)	0.0025 (6)	0.0021 (6)	−0.0016 (6)
C7	0.0525 (8)	0.0459 (7)	0.0519 (8)	0.0048 (7)	0.0102 (7)	0.0047 (6)
C6	0.0519 (9)	0.0532 (8)	0.0523 (8)	0.0089 (7)	0.0044 (7)	0.0069 (6)
C10	0.0448 (9)	0.0483 (8)	0.0641 (9)	−0.0039 (6)	−0.0012 (7)	−0.0083 (7)
C8	0.0562 (9)	0.0560 (9)	0.0532 (9)	0.0104 (7)	−0.0019 (7)	−0.0021 (7)
C2	0.0540 (9)	0.0503 (8)	0.0644 (10)	0.0024 (7)	0.0110 (8)	0.0020 (7)
C12	0.0607 (10)	0.0598 (9)	0.0670 (10)	0.0024 (8)	0.0146 (8)	−0.0071 (8)
C9	0.0535 (9)	0.0523 (9)	0.0805 (11)	0.0008 (7)	0.0022 (8)	−0.0051 (8)
C11	0.0675 (11)	0.0570 (9)	0.0665 (10)	0.0020 (8)	0.0128 (9)	0.0029 (7)
C5	0.0690 (11)	0.0868 (12)	0.0527 (9)	0.0083 (10)	0.0066 (8)	0.0126 (8)
C3	0.0629 (11)	0.0631 (10)	0.0865 (13)	−0.0018 (8)	0.0170 (10)	0.0180 (9)
C4	0.0742 (12)	0.0940 (14)	0.0699 (12)	0.0043 (11)	0.0188 (10)	0.0333 (10)
C1	0.0695 (12)	0.0710 (12)	0.1064 (15)	−0.0183 (9)	0.0202 (11)	−0.0169 (10)

### Geometric parameters (Å, º)

**Table:**

O2—C7	1.3438 (17)	C2—C3	1.375 (2)
O2—H2	0.858 (13)	C12—C11^i^	1.386 (2)
O1—C2	1.3668 (19)	C12—H12	0.9300
O1—C1	1.418 (2)	C9—H9A	0.9700
N1—C8	1.2717 (19)	C9—H9B	0.9700
N1—C9	1.463 (2)	C11—H11	0.9300
C7—C6	1.396 (2)	C5—C4	1.361 (3)
C7—C2	1.403 (2)	C5—H5	0.9300
C6—C5	1.396 (2)	C3—C4	1.388 (3)
C6—C8	1.451 (2)	C3—H3	0.9300
C10—C12	1.374 (2)	C4—H4	0.9300
C10—C11	1.379 (2)	C1—H1A	0.9600
C10—C9	1.511 (2)	C1—H1B	0.9600
C8—H8	0.9300	C1—H1C	0.9600
			
C7—O2—H2	104.1 (13)	C10—C9—H9A	109.6
C2—O1—C1	117.26 (13)	N1—C9—H9B	109.6
C8—N1—C9	118.17 (14)	C10—C9—H9B	109.6
O2—C7—C6	122.05 (13)	H9A—C9—H9B	108.1
O2—C7—C2	118.20 (14)	C10—C11—C12^i^	120.95 (15)
C6—C7—C2	119.75 (13)	C10—C11—H11	119.5
C7—C6—C5	119.45 (15)	C12^i^—C11—H11	119.5
C7—C6—C8	120.53 (13)	C4—C5—C6	120.35 (17)
C5—C6—C8	120.02 (15)	C4—C5—H5	119.8
C12—C10—C11	118.18 (14)	C6—C5—H5	119.8
C12—C10—C9	121.52 (14)	C2—C3—C4	120.71 (17)
C11—C10—C9	120.30 (15)	C2—C3—H3	119.6
N1—C8—C6	122.40 (14)	C4—C3—H3	119.6
N1—C8—H8	118.8	C5—C4—C3	120.38 (16)
C6—C8—H8	118.8	C5—C4—H4	119.8
O1—C2—C3	125.21 (15)	C3—C4—H4	119.8
O1—C2—C7	115.45 (13)	O1—C1—H1A	109.5
C3—C2—C7	119.34 (16)	O1—C1—H1B	109.5
C10—C12—C11^i^	120.88 (14)	H1A—C1—H1B	109.5
C10—C12—H12	119.6	O1—C1—H1C	109.5
C11^i^—C12—H12	119.6	H1A—C1—H1C	109.5
N1—C9—C10	110.42 (13)	H1B—C1—H1C	109.5
N1—C9—H9A	109.6		
			
O2—C7—C6—C5	178.60 (14)	C11—C10—C12—C11^i^	−0.1 (3)
C2—C7—C6—C5	−1.3 (2)	C9—C10—C12—C11^i^	179.25 (15)
O2—C7—C6—C8	−1.9 (2)	C8—N1—C9—C10	−102.38 (16)
C2—C7—C6—C8	178.27 (13)	C12—C10—C9—N1	−108.69 (17)
C9—N1—C8—C6	178.54 (13)	C11—C10—C9—N1	70.69 (19)
C7—C6—C8—N1	3.8 (2)	C12—C10—C11—C12^i^	0.1 (3)
C5—C6—C8—N1	−176.65 (14)	C9—C10—C11—C12^i^	−179.26 (15)
C1—O1—C2—C3	0.4 (2)	C7—C6—C5—C4	0.2 (2)
C1—O1—C2—C7	−179.17 (14)	C8—C6—C5—C4	−179.33 (15)
O2—C7—C2—O1	0.7 (2)	O1—C2—C3—C4	−179.08 (16)
C6—C7—C2—O1	−179.47 (13)	C7—C2—C3—C4	0.5 (2)
O2—C7—C2—C3	−178.96 (14)	C6—C5—C4—C3	1.2 (3)
C6—C7—C2—C3	0.9 (2)	C2—C3—C4—C5	−1.6 (3)

Symmetry code: (i) −*x*+2, −*y*+1, −*z*+1.

### Hydrogen-bond geometry (Å, º)

**Table:**

*D*—H···*A*	*D*—H	H···*A*	*D*···*A*	*D*—H···*A*
O2—H2···N1	0.86 (1)	1.79 (2)	2.5877 (18)	154 (2)
C8—H8···O2^ii^	0.93	2.51	3.410 (2)	162

Symmetry code: (ii) *x*+1/2, −*y*+1/2, *z*+1/2.
